# Moderating Effects of Social Value Orientation on the Effect of Social Influence in Prosocial Decisions

**DOI:** 10.3389/fpsyg.2016.00952

**Published:** 2016-06-21

**Authors:** Zhenyu Wei, Zhiying Zhao, Yong Zheng

**Affiliations:** ^1^Center for Studies of Education and Psychology of Ethnic Minorities in Southwest China, Southwest UniversityChongqing, China; ^2^Faculty of Psychology, Southwest UniversityChongqing, China; ^3^Key Laboratory for NeuroInformation of Ministry of Education, School of Life Science and Technology, University of Electronic Science and Technology of ChinaChengdu, China; ^4^Key Laboratory of Cognition and Personality (MOE), Southwest UniversityChongqing, China

**Keywords:** social value orientation, social influence, prosocial decision, trust, generosity

## Abstract

Prosocial behaviors are susceptible to individuals’ preferences regarding payoffs and social context. In the present study, we combined individual differences with social influence and attempted to discover the effect of social value orientation (SVO) and social influence on prosocial behavior in a trust game and a dictator game. Prosocial behavior in the trust game could be motivated by strategic considerations whereas individuals’ decisions in the dictator game could be associated with their social preference. In the trust game, prosocials were less likely than proselfs to conform to the behavior of other group members when the majority of group members distrusted the trustee. In the dictator game, the results of the three-way ANOVA indicated that, irrespective of the type of offer, in contrast to proselfs, prosocials were influenced more by others’ generous choices than their selfish choices, even if the selfish choices were beneficial to themselves. The overall results demonstrated that the effect of social influence appears to depend on individuals’ SVO: that is, prosocials tend to conform to prosocial rather than proself behaviors.

## Introduction

People often face mixed-motive social dilemmas in which their self-interest is at variance with what is best for their community (Balliet et al., 2009). Previous studies have shown that people differ in fundamental ways in how they approach and interact in social dilemmas (Van Lange et al., 2013a,b). Social value orientation (SVO) has been defined as a personal trait that reflects how people resolve social dilemmas (Messick and McClintock, 1968; Kelley and Stahelski, 1970; Kuhlman and Marshello, 1975; Liebrand, 1984; McClintock and Liebrand, 1988; Van Lange, 1999). The implications of individual differences in SVO refer to people’s self-regarding versus other-regarding preferences (Van Lange, 2000). The most common manner of assessing SVO is by means of decomposed games (Liebrand, 1984; McClintock and Liebrand, 1988). Researchers have noted that three SVOs are common (Messick and McClintock, 1968): individuals can be classified as prosocials, individualists, and competitors. Prosocials are defined as individuals who attempt either to maximize the welfare of others or to choose joint gain. Individualists prefer to maximize their own welfare, showing little concern with others’ outcomes. Finally, competitors attempt to maximize the difference between their own welfare and others’ outcomes (Messick and McClintock, 1968; Kuhlman and Marshello, 1975; Van Lange, 1999). Because competitors show non-cooperative behavior similar to individualists’ and the proportion of competitors is quite small, previous studies have combined individualists and competitors into a category called “proselfs” (Van Lange and Liebrand, 1991; Van Lange et al., 1998; Bogaert et al., 2008).

Previous studies have attempted to link SVO with individuals’ behavior in prosocial decisions (McClintock and Allison, 1989; Van Lange et al., 1998, 2007; Kanagaretnam et al., 2009). Behavior is considered to be prosocial when it benefits others (Batson and Powell, 2003; Twenge et al., 2007; Piff et al., 2010; Zaki and Mitchell, 2011). Most cultures encourage or even require prosocial behavior because it is vital to the social system. People often perform prosocial behaviors because doing so enables them to belong to their community or society and to enjoy the social reward (i.e., a good reputation). Prior studies have demonstrated that prosocials are more generous in their helping responses than proselfs and more engaged in donating money to organizations aimed at helping the poor and the ill (McClintock and Allison, 1989; Van Lange et al., 2007). Prosocials also exhibit greater trust than individualists in the trust game (Kanagaretnam et al., 2009).

During the past decade, researchers have been interested in understanding how SVO interacts with features of a social situation to predict behavior (Balliet et al., 2009). Social influence plays an important role in our daily lives. We live in a highly complex social environment where social information continuously affects our perception and decision-making. Previous studies have shown that individuals tend to change their opinions and behaviors in order to align with group norms (Cialdini and Goldstein, 2004). This phenomenon, known as “social conformity”, refers to the action of changing one’s initial choices or opinions to match those of the group majority (Turner, 1991). Following the work of Asch (1951), psychologists have extensively examined the causes and underlying mechanisms of social conformity. Three motivations relate to conforming behavior: a desire to be correct, a desire to obtain social approval from others, and a desire to maintain a positive self-concept (Cialdini and Goldstein, 2004). Previous studies have shown that social influence can motivate people to behave prosocially (Shang and Croson, 2009; Nook et al., in press). However, they leave important questions unanswered because they say little about the individual differences in prosocial conformity. Some studies have demonstrated that conformity behavior could be modulated by personality traits (Steiner and Vannoy, 1966; DeYoung et al., 2002). From this perspective, SVO, which has been defined as a personal trait that reflects individuals’ social preferences, could affect individuals’ willingness to follow the majority in prosocial behavior. To address this question, we designed two tasks to investigate how SVO influences individuals’ conformity behaviors in trusting behavior and generous behavior.

In Study 1, we investigated the interaction between SVO and social influence in trusting behavior using the trust game. There are two players in the original trust game: an investor and a trustee (Berg et al., 1995). Both players are endowed with $10. First, the investor decides whether to give the endowment to the trustee. Then, the amount given is multiplied by the experimenter. Finally, the trustee chooses whether to keep the amount he/she received or pass any portion of the money back to the investor. The amount passed by the investor is used to capture trust. Trust refers to a willingness to bet that the other will reciprocate a risky move even at a cost to themselves (Camerer, 2003). Prosocial behavior in the trust game could emanate from strategic considerations (Espín et al., 2016). In the present study, we developed a variant of the trust game in which participants, who were able to see other group members’ choices before making a decision, were asked to decide whether to send the endowment to a stranger or to keep the endowment. We predicted that participants’ rate of trust in the trust game is dependent on their SVO. Compared with proselfs, prosocials should be more trusting in the trust game (Kanagaretnam et al., 2009). In addition, we hypothesized that the choices of the majority would affect participants’ trusting behavior: that is, subjects would trust the trustee when they see that the majority of the group does so. Further, previous studies have shown that conformity behavior could be affected by personality traits (Steiner and Vannoy, 1966; DeYoung et al., 2002). With the assumption that SVO, which has been defined as a fundamental personal trait that reflects how people resolve social dilemmas, could influence individuals’ decisions in the trust game (Kanagaretnam et al., 2009), we expected that the effect of social influence in the trust game would be modulated by SVO.

In Study 2, we were interested in the interaction between SVO and social influence in the dictator game. We used a modified dictator game, which was designed by Zaki and Mitchell (2011), to investigate the effect of social influence on generous behavior, free of strategic considerations. Participants made iterated choices about whether to allocate varying amounts of money to themselves or to another person (see *Experimental design and procedure in Study 2* for details). This task yields a behavioral measure of generosity (giving to the receiver at a cost to one’s self) (Zaki and Mitchell, 2011). Participants’ decision in this task could be motivated by their social preference rather than strategic considerations because the second player is passive. We assumed that participants’ choices would be dependent on their SVO: that is, compared with proselfs, prosocials would tend to make more generous choices in the dictator game. We also hypothesized that the choices of the majority would affect subjects’ choices: that is, subjects would make more generous choices when they saw that the majority of the group allocated money to the receiver. In the end, as prosocials show a natural willingness to help others and they are more generous than proselfs (McClintock and Allison, 1989; Van Lange et al., 2007), we predicted that the effect of social influence in the dictator game would be modulated by SVO and that prosocials might be less likely to be influenced by the selfish choices of group members.

## Study 1

### Materials and Methods

#### Participants

One hundred thirty-six healthy right-handed participants completed study 1. All were native Mandarin speakers, with no neurological or psychological disorders, and with normal color vision. Written informed consent was obtained after detailed explanation of the experiment. This study was conducted in accordance with the Declaration of Helsinki and approved by the Ethics Committee of Southwest University.

#### Measurement of Social Value Orientation

We used a questionnaire including a series of nine decomposed games to assess a participant’s SVO (Van Lange and Kuhlman, 1994; Van Lange et al., 1997). This questionnaire is an efficient and easy-to-administer instrument (Van Lange et al., 1997). Subjects were classified as prosocial, individualistic, or competitive if at least six of nine decisions were consistent with a particular value orientation (Van Lange and Kuhlman, 1994; Van Lange et al., 1997). One hundred sixteen participants fell into one of three SVO. We identified 52 prosocials (35 females), 56 individualists (23 females) and 8 competitors (1 females). Following prior research on SVO, we combined the individualists and competitors to form a group of proselfs (Van Lange and Liebrand, 1991; Van Lange et al., 1998).

#### Experimental Design and Procedure

After arrived at the laboratory, participants were told that they would perform the experiment with four other participants, who were in separate rooms, but that they would see the choices of the other group members on the computer screen during the experiment. In the experiment, participants would play an on-line monetary game as an investor independently with 70 different strangers (trustees). The strangers were randomly selected from the university and played the game with participants through a local network. Other group members also did not know anything about trustees.

In each trial, both players were endowed with ¥2. The investor was restricted to the options of keeping the endowment or sending all ¥2. If the investor decided to send ¥2 to the trustee, this money would be tripled. Then the trustee was restricted to either send nothing back or send half of the tripled amount back (¥3). However, the investor would not know the outcome (i.e., trustee’s choice) during the task. Subjects were told that they will receive ¥10 for participating in the experiment plus the additional money earned from ten of their decisions, chosen at random, during the trust game. Actually, subjects earned a show-up fee (¥15) and a bonus (¥4). We asked participants whether s/he believed the existence of trustees and group members after they finished the task. Six participants reported that they did not believed the existence of trustees. Therefore, their data were excluded in the analysis.

The hypotheses of this study were tested in a 2 × 3 (SVO: Prosocial Orientation vs. Proself Orientation × Social Influence: No influence vs. Trust influence vs. Distrust influence) factorial design. The experiment contained one block (70 trials). The duration of a trial is approximately 11 s. In 10 of the trials, two peers decided to send the endowments to the trustee while the other two peers decided to keep the endowments. These trials were not included in the final analysis because they were used solely to maintain believability of the interaction between participant and the four peers. In one-third of the remaining trials (20 trials), the group’s choices were hidden from the subject (the no information, or baseline condition; we told participants that they would not see their peers’ choices in these trials because the decisions in these trials were not made by all of the four other members). In this situation, they would see four “ × ” symbols. For the 20 trials of the trust influence condition, three or four group members decided to send the endowments to the trustee. For the 20 trials of the distrust influence condition, one or none of the group members decided to send the endowments to the trustee. These trials were presented in a random order.

**FIGURE 1:**
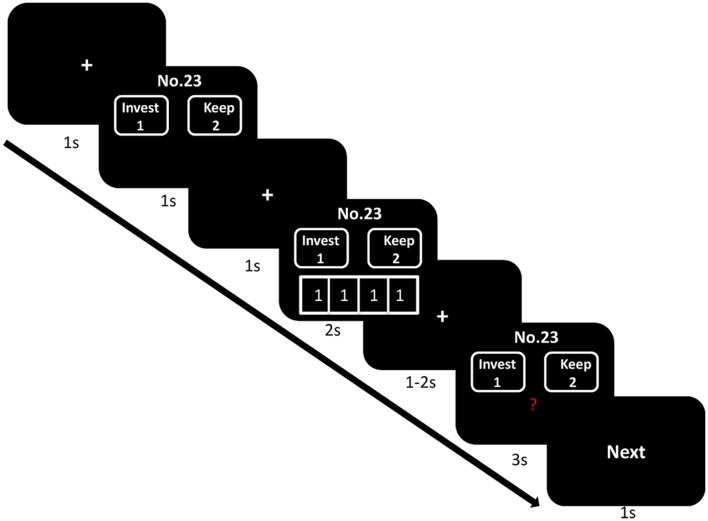
**Demonstration of sequence of events in a trial (take trust influence as an illustration)**.

Participants then received details about the procedure of the experiment. At the beginning of each trial, the participants were presented with a fixation point for a 1s duration. The offer would be shown on the screen for 1 s, followed by a fixation point for duration of 1 s. They could see the number of the trustee in the top of the offer screen. Then the choices of group members would be presented for 2 s under the offer, followed by a fixation point for duration of 1–2 s. Subsequently, the decision phase was shown on the screen for 3 s. Participant used the index and middle fingers of their right hand to respond to the offer by pressing keyboard (“1” to invest and “2” to keep the endowment). In the end, the word “next” displayed for 1s, which indicated that the next trial was about to begin. The sequence of events in a trial is illustrated in **Figure 1**. Before performing the task, participants completed a training session. We told participants that the computer used to conduct the pre-experiment training was not connected to the local network, therefore the choices of the other group members would remain hidden. A PC running E-Prime 2.0 was used to display the stimuli and acquire the responses of the participants.

### Results

Trials in which the subjects did not respond in the decision stage were excluded from further data analyses. 5.3% of total trials were rejected to enter the following data analyses. Social influence effect was measured by the rate of trust of participants. A 2 (SVO: proselfs, prosocials) × 3 (social influence: trust, distrust, baseline) repeated measure ANOVA revealed a significant main effect of the factor social influence, *F*(2,113) = 14.31, *p* < 0.001. Participants trusted the trustee at a significantly higher rate in the trust condition (*M* = 0.69, *SD* = 0.3) than in the distrust condition (*M* = 0.43, *SD* = 0.32) and baseline (*M* = 0.56, *SD* = 0.26). The main effect of SVO was significant, *F*(1,114) = 10.74, *p* < 0.001. Prosocial individuals (*M* = 0.62, *SD* = 0.31) trusted the trustee at a significantly higher rate than proself individuals (*M* = 0.51, *SD* = 0.31).

**FIGURE 2:**
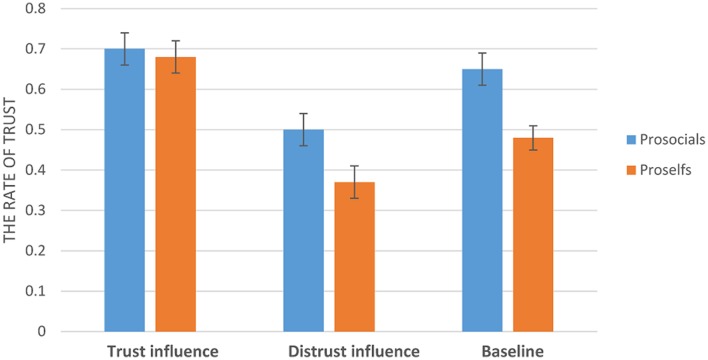
**The rate of trust (Error bars represent standard errors of the mean)**.

The interaction between SVO and social influence was significant, *F*(2,113) = 4.23, *p* < 0.05 (**Figure 2**). The results indicated that prosocial individuals (*M* = 0.65, *SD* = 0.24) trusted the trustee at a significant higher rate than proself individuals (*M* = 0.48, *SD* = 0.25) in the baseline condition, *F*(1,114) = 13.91, *p* < 0.001. In addition, prosocial individuals also (*M* = 0.5, *SD* = 0.33) trusted the trustee at a significant higher rate than proself individuals (*M* = 0.37, *SD* = 0.3) in the distrust condition, *F*(1,114) = 4.49, *p* < 0.05. The difference between prosocials (*M* = 0.7, *SD* = 0.31) and proselfs (*M* = 0.68, *SD* = 0.29) in the trust condition was not significant, *F*(1,114) = 0.1, *p* = 0.75.

### Discussion

A prior study found that genetics explain about 20% of the cross-sectional variation in trust game behavior (Cesarini et al., 2008), thus suggesting stable individual differences in trust. Our results, like those of Kanagaretnam et al. (2009), suggest that SVO may partially underlie such individual differences. However, the findings of Cesarini et al. (2008) also indicate that about 80% of variation must be explained by unknown environmental factors (Ahern et al., 2014). According to the present findings, social conformity might be one such factor since individuals’ behavior in the trust game could indeed be influenced by the opinions of peers (as in other environments; see Cialdini and Goldstein, 2004). The present study showed that prosocials were less likely than proselfs to conform to group members when the majority of group members did not trust the trustee.

Prosocials tend to consider the impact of their behavior on others and strive to maximize joint outcomes (De Cremer and Van Lange, 2001). They prefer to seek win-win situations in a disagreement (Van Lange et al., 1997). In contrast to prosocials, proselfs strive to maximize their own outcomes. Therefore, prosocials show a higher level of prosocial behavior than proselfs in the trust game (Kanagaretnam et al., 2009). In the present study, prosocials showed a lower level of conformity behavior than proselfs when group members distrusted the trustee. We infer that prosocials are less influenced by group members’ distrust behavior because they are naturally prosocial and trusting individuals. In this vein, it might be argued that peers’ choices in the trust game serve as a cue of expected trustworthiness, which could affect individuals’ emotional systems in decision making. Because participants were asked to make decisions under time pressure, the emotional reactions could guide their decisions. As a previous study showed, some people trust the trustee due to strategic self-interest whereas other people trust the trustee because of social efficiency reasons (Espín et al., 2016). Prosocials care more about the social efficiency whereas proselfs tend to be self-interested. Therefore, prosocials still trust the trustee when they perceive that the trustee will not reciprocate (i.e., the distrust condition).

## Study 2

### Materials and Methods

#### Participants

One hundred three healthy right-handed participants completed study 2. All were native Mandarin speakers, with no neurological or psychological disorders, and with normal color vision. Written informed consent was obtained after detailed explanation of the experiment. This study was conducted in accordance with the Declaration of Helsinki and approved by the Ethics Committee of Southwest University.

#### Measurement of Social Value Orientation

We used a questionnaire including a series of nine decomposed games to assess a participant’s SVO (Van Lange and Kuhlman, 1994; Van Lange et al., 1997). Subjects were classified as prosocial, individualistic, or competitive if at least six of nine decisions were consistent with a particular value orientation (Van Lange and Kuhlman, 1994; Van Lange et al., 1997). Ninety-five participants fell into one of three SVO. We identified 47 prosocials (29 females), 42 individualists (23 females), and 6 competitors. Following prior research on SVO, we combined the individualists and competitors to form a group of proself individuals (Van Lange and Liebrand, 1991; Van Lange et al., 1998).

#### Experimental Design and Procedure

The hypotheses of this study were tested in a 2 × 2 × 3 (SVO: Prosocial Orientation vs. Proself Orientation × Offer Type: Selfish vs. Generous × Social Influence: No influence vs. Selfish influence vs. Generous influence) factorial design. Each trial began with two monetary offers, one associated with the participant and the other with the receiver. Participants made iterated choices about whether to allocate varying amounts of money to themselves or to the receiver. For example, if the offer assigned ¥1.00 to the participant and ¥3.00 to the receiver, participants should choose between ¥1.00 for themselves and ¥3.00 for the receiver. The amounts that each person stood to gain varied across trials but always adhered to one of a set of six ratios specifying the relationship between the self vs. other monetary amounts: 3:1, 2:1, 3:2, 4:3, 5:4, and 1:1. Each ratio could produce two relationships between the amounts that the participant and the receiver stood to gain. Thus there were eleven ratios in present experiment (3:1, 2:1, 3:2, 4:3, 5:4, 1:1, 4:5, 3:4, 2:3, 1:2, and 1:3). For each trial, a random value between ¥0.00 and ¥3.00 was chosen, and a second value was determined by transforming the first value according to the ratio that applied during that trial. For example, if the amount of ¥1.00 was selected and the ratio was 2:1, the other one was ¥0.5. The maximum amount that either the participant or receiver stood to gain in one trial was ¥9.00.

The experiment contained 120 trials. On average, a trial lasted 13 s. There were five types of offers in the present study. If the ratio were larger than 1:1 (e.g., 2:1), the offer was a selfish offer. If the ratio were smaller than 1:1 (e.g., 1:2), the offer was a generous offer. If the ratio was 1:1, the offer was an equal offer. Besides, we also added “pure-self” and “pure-other” offers in the experiment. During pure-self trials the participant was presented with offers of a non-zero amount of money (e.g., ¥1.00) for herself/himself and ¥0.00 for the receiver, while in the pure-other condition, the participant was presented with offers of ¥0.00 for herself/himself and a non-zero amount of money for the receiver. Finally, we also added non-reward trials in which participants chose between ¥0.00 for herself/himself and ¥0.00 for the receiver. Overall, there were fifty selfish offers, fifty generous offers, ten equal offers, ten pure-self offers, ten pure-other offers and ten non-reward offers. The selfish offer condition and generous offer condition each comprised 15 selfish influence trials, 15 generous influence trials, 15 baseline trials and 5 mediate influence trials (these trials were not included in the final analysis because they were used solely to maintain believability of the interaction between participant and the four peers). We only included trials in which the participant and receiver stood to gain unequal, non-zero amounts of money.

When participants arrived at the laboratory, they were told individually that they would participate in the experiment with another four subjects, who were in separate rooms. In the experiment, they would independently play a monetary game with a human receiver, who would be in the other room. Group members and participants knew nothing about each other and they were told that they would also not meet each other after the experiment. Participants were told that they would be making repeated decisions about whether to allocate money to themselves, or to the receiver. Five of their decisions, chosen at random by the system, would be enacted and added to the final payment. To minimize the influence of reputation motives on the participant’s choices, participants were told that the receiver would not know that the participant had completed the distribution game, and that the additional compensation would simply be included in the receiver’s payment after the experiment. The participant could observe the choices of the other four group members through a local network on the computer during the experiment, but group members would not know the participant’s choices. In addition, because participants used different computers, and because the order of offer presentation is random, they would sometimes not see the choices of group members if a group member had not responded to the offer. In this situation, they would see four “ × ” symbols. These trials were classified as the baseline condition. These instructions allowed participants to believe in the existence of the other group members. We asked participants whether s/he believed the existence of group members and the receiver after they finished the task. One participant reported that he did not believed the existence of group members and the receiver. Therefore, his data were excluded in the analysis.

**FIGURE 3:**
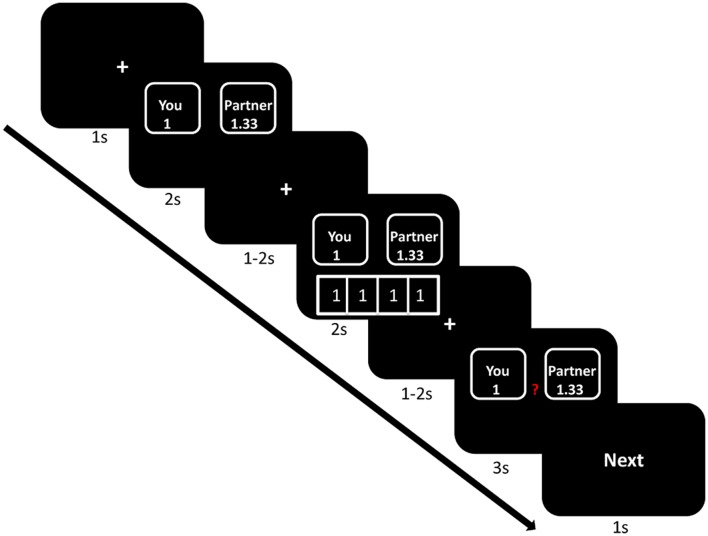
**Demonstration of sequence of events in a trial (take generous offer and selfish influence as an illustration)**.

Participants then received instructions about how the experiment would proceed. At the beginning of each trial, the participants were presented with a fixation point for 1 s. The offer would be shown on the screen for 2 s, followed by a fixation point for duration of 1–2 s. Then, the choices of group members would be presented for 2 s underneath the offer, followed by a fixation point that would last for duration of 1–2 s. In the end, the decision phase was shown on the screen for 3 s. Participants used the index and middle fingers of their right hand to respond to the offer by pressing one of the two buttons on the keyboard (“1” to allocate to self and “2” to allocate to the receiver). The decision phase was followed by the word “Next”, which was displayed for 1 s and indicated that the next trial was about to begin. The sequence of events in a trial is illustrated in **Figure 3**. Before performing the task, participants completed a training session. We told participants that the computer used to conduct the pre-experiment training was not connected to the local network, therefore the choices of the other group members would remain hidden. A PC running E-Prime 2.0 was used to display the stimuli and acquire the responses of the participants.

### Results

Trials in which the subjects did not respond in the decision stage were excluded from further data analyses. 4.6% of total trials were rejected to enter the following data analyses.

Social influence effect was measured by the rate of allocate money to the receiver. A 2 (SVO: proselfs, prosocials) × 2 (offer: selfish, generous) × 3 (social influence: selfish, generous, baseline) repeated measure ANOVA revealed a significant main effect of the factor social influence, *F*(2,92) = 21.27, *p* < 0.001. Participants allocated money to receiver at a significantly higher rate in the generous influence condition (*M* = 0.5, *SD* = 0.35) than in the selfish influence condition (*M* = 0.39, *SD* = 0.34). The main effect of offer was significant, *F*(1,93) = 93.87, *p* < 0.001. Participants allocated money to the receiver at a significantly higher rate in generous offer condition (*M* = 0.61, *SD* = 0.31) than in selfish offer condition (*M* = 0.24, *SD* = 0.26). This result is consistent with a previous study (Zaki and Mitchell, 2011). The main effect of SVO was close to significance (*M*_proselfs_ = 0.39, *SD*_proselfs_ = 0.22; *M*_prosocials_ = 0.45, *SD*_prosocials_ = 0.23), *F*(1,93) = 3.47, *p* = 0.067.

The interaction between offer and social influence was significant, *F*(2,92) = 8.49, *p* < 0.001. The result indicated that participants allocated money to the receiver at a significantly higher rate (*M* = 0.7, *SD* = 0.3) in generous offer-generous influence condition than in generous offer-selfish influence condition (*M* = 0.59, *SD* = 0.3), *p* < 0.001, and in generous offer-baseline condition (*M* = 0.54, *SD* = 0.3), *p* < 0.01. In the selfish offer condition, participants allocated money to the receiver at a significantly higher rate in the generous influence condition (*M* = 0.3, *SD* = 0.25) than in the selfish influence condition (*M* = 0.19, *SD* = 0.25), *p* < 0.001, and in baseline (*M* = 0.22, *SD* = 0.25), *p* < 0.001. The difference between selfish influence condition and baseline condition was not significant, *p* = 0.081. The interaction between SVO and social influence was not significant, *F*(2,92) = 3.47, *p* = 0.478.

**FIGURE 4:**
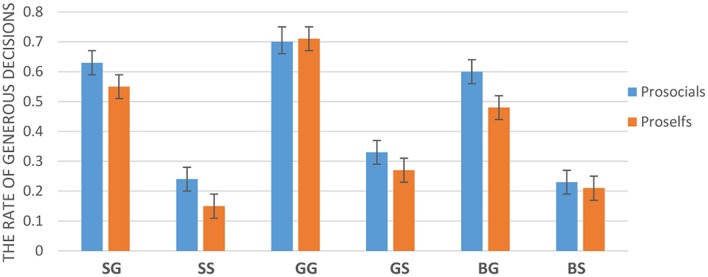
**The rate of generous decisions (SG: selfish influence-generous offer; SS: selfish influence-selfish offer; GG: generous influence-generous offer; GS: generous influence-selfish offer; BG: baseline-generous offer; BS: baseline-selfish offer**. Error bars represent standard errors of the mean).

The interaction between SVO, offer type and social influence was significant, *F*(2,92) = 4.97, *p* < 0.01 (**Figure 4**). Regardless of the type of offer, proselfs allocated money to the receiver at a significantly higher rate in the generous influence condition (*M*_selfishoffer_ = 0.27, *SD*_selfishoffer_ = 0.19; *M*_generousoffer_ = 0.71, *SD*_generousoffer_ = 0.26) than in selfish influence condition (*M*_selfishoffer_ = 0.15, *SD*_selfishoffer_ = 0.16, *p* < 0.001; *M*_generousoffer_ = 0.55, *SD*_generousoffer_ = 0.25, *p* < 0.001), and in baseline condition (*M*_selfishoffer_ = 0.21, *SD*_selfishoffer_ = 0.2, *p* < 0.05; *M*_generousoffer_ = 0.48, *SD*_generousoffer_ = 0.26, *p* < 0.001). For prosocials, they allocated money to the receiver at a significantly higher rate in generous influence condition (*M* = 0.7, *SD* = 0.35) than in the baseline (*M* = 0.6, *SD* = 0.33) when the offer is generous offer, *p* < 0.05. However, the difference between selfish influence condition (*M* = 0.63, *SD* = 0.34) and baseline was not significant, *p* = 0.268. In addition, the difference between selfish influence condition and generous influence condition was also not significant, *p* = 0.126. In the selfish offer condition, prosocials allocated money to the receiver at a significantly higher rate in the generous influence condition (*M* = 0.33, *SD* = 0.31) than in selfish influence condition (*M* = 0.24, *SD* = 0.32, *p* < 0.01) and in baseline condition (*M* = 0.23, *SD* = 0.3, *p* < 0.001). The difference between selfish influence condition and baseline was not significant, *p* = 0.926.

### Discussion

Study 2 set out to investigate the effects of SVO and social influence in generous decisions. People often change their decisions and judgments to conform to normative group behavior (Cialdini and Goldstein, 2004; Klucharev et al., 2009; Wei et al., 2013). The present study showed that individuals’ generous decisions can be influenced by the group members’ choices; however, this effect can be modulated by individuals’ SVO. Results of the three-way ANOVA showed that no matter the offers were selfish or generous, proselfs were influenced by others’ selfish choices and generous choices. However, when it comes to prosocials, they were influenced by others’ generous choices rather than their selfish choices.

Generosity is defined as helping another at a cost to oneself; therefore, generosity is a kind of prosocial behavior (Zak et al., 2007). Prosocials have a stable preference for maximizing joint outcomes, but proselfs prefer to maximize their own benefits (Van Lange, 2000). Additionally, prior studies have demonstrated that prosocials are more generous in their helping responses than proselfs and more engaged in donating money to organizations aimed at helping the poor and the ill (McClintock and Allison, 1989; Van Lange et al., 2007). We infer that selfish choices are conflict with prosocials’ social preference and prosocials know that selfish behavior is not encouraged by social norms. Therefore, in both offer conditions, prosocials were influenced by others’ generous choices rather than their selfish choices, even if the selfish choices were beneficial to themselves.

## General Discussion

Social value orientation is regarded as a stable personality trait that reflects how people evaluate outcomes for self and others (Messick and McClintock, 1968). Individual SVO can determine and predict individuals’ choice behavior in a wide variety of decisions (Messick and McClintock, 1968; Kuhlman and Marshello, 1975; Van Lange, 1999), including prosocial decisions (McClintock and Allison, 1989; Van Lange et al., 1998, 2007; Kanagaretnam et al., 2009). According to previous studies, prosocials tend to trust others, and they are more generous than proselfs (McClintock and Allison, 1989; Van Lange et al., 2007; Kanagaretnam et al., 2009). In the present study, in agreement with previous ones, we found that people tend to conform to the choices of group members in prosocial decisions. However, our study also found that individuals’ SVO could modulate the effect of social influence in prosocial decisions. Relative to proselfs, prosocials were less likely to conform to proself behaviors. We infer that prosocials know that proself behavior is not accepted by general social norms and they can resist the proself choices of other group members. Proselfs, as well, know that prosocial behavior is encouraged by social norms. Therefore, they would experience group pressure when they realized that the majority was choosing the prosocial option (Asch, 1951; Strickland and Crowne, 1962; Becker et al., 1964) and would then be more likely to conform to the choices of group members, even when these choices conflicted with their own preferences.

## Conclusion

Prior experimental studies have provided evidence that prosocial behaviors are susceptible to individuals’ preferences for payoffs and social context (McCabe et al., 2003; Boone et al., 2008; Declerck et al., 2010). In the present studies, we combined individual differences with social influence in an attempt to discover the effect of SVO and social influence on prosocial behavior in the trust game and the dictator game. Our results extend our current understanding of prosocial conformity by showing that the effect of social influence on prosocial behavior depends on a person’s SVO. Prosocials tend to follow prosocial choices rather than proself behaviors. Prosocials have a natural willingness to behave prosocially and they know that prosocial behavior is encouraged by social norms (McClintock and Allison, 1989; Van Lange et al., 2007). Therefore, they can resist the proself influence that conflicts with their own social preference.

## Author Contributions

Conceived and designed the experiments: ZW and YZ. Program the task: ZW and ZZ. Performed the experiments: ZW. Analyzed the data: ZW. Wrote the paper: ZW, ZZ, and YZ.

## Conflict of Interest Statement

The authors declare that the research was conducted in the absence of any commercial or financial relationships that could be construed as a potential conflict of interest. The reviewer GG and handling Editor declared their shared affiliation, and the handling Editor states that the process nevertheless met the standards of a fair and objective review.
